# Analysis and comprehensive comparison of PacBio and nanopore-based RNA sequencing of the *Arabidopsis* transcriptome

**DOI:** 10.1186/s13007-020-00629-x

**Published:** 2020-06-12

**Authors:** Jiawen Cui, Nan shen, Zhaogeng Lu, Guolu Xu, Yuyao Wang, Biao Jin

**Affiliations:** 1 College of Horticulture and Plant Protection, Yangzhou University, Yangzhou, 225009 China; 2 Biomarker Technologies Corporation, Beijing, 101300 China

**Keywords:** Plant transcriptome, Third-generation sequencing, PacBio, Nanopore, RNA-Seq, Single-molecule sequencing

## Abstract

**Background:**

The number of studies using third-generation sequencing utilising Pacific Biosciences (PacBio) and Oxford Nanopore Technologies (ONT) is rapidly increasing in many different research areas. Among them, plant full-length single-molecule transcriptome studies have mostly used PacBio sequencing, whereas ONT is rarely used. Therefore, in this study, we examined ONT RNA sequencing methods in plants. We performed a detailed evaluation of reads from PacBio, Nanopore direct cDNA (ONT Dc), and Nanopore PCR cDNA (ONT Pc) sequencing including characteristics of raw data and identification of transcripts. In addition, matched Illumina data were generated for comparison.

**Results:**

ONT Pc showed overall better raw data quality, whereas PacBio generated longer read lengths. In the transcriptome analysis, PacBio and ONT Pc performed similarly in transcript identification, simple sequence repeat analysis, and long non-coding RNA prediction. PacBio was superior in identifying alternative splicing events, whereas ONT Pc could estimate transcript expression levels.

**Conclusions:**

This paper made a comprehensive comparison of PacBio and nanopore-based RNA sequencing of the *Arabidopsis* transcriptome, the results indicate that ONT Pc is more cost-effective for generating extremely long reads and can characterise the transcriptome as well as quantify transcript expression. Therefore, ONT Pc is a new cost-effective and worthwhile method for full-length single-molecule transcriptome analysis in plants.

## Background

Current sequencing-based transcriptomic analyses (RNA-Seq) using the massive throughput of next-generation sequencing platforms have enabled us to build a picture of the active transcriptional patterns within organisms. Among these analyses, short-read RNA-Seq (mainly using Illumina technology) has been used for over a decade. The numbers of reads output by Illumina sequencers could accurately quantify the highly expressed genes. However, because Illumina sequencers are appropriate only for short read-length sequencing, we must fragment RNA or cDNA during sample preparation. Thus, the read length is the major limitation in short-read RNA-Seq which would cause the loss of some information from the original full-length transcripts, and therefore it is hard to analyse several aspects of co/post-transcriptional processing events.

With the rapid development of sequencing technology, long-read sequencing platforms, including Pacific Biosciences (PacBio) [1] and Oxford Nanopore Technologies (ONT) [2], which have the capability to sequence entire cDNA molecules end-to-end, are available. These two platforms have increased read lengths considerably in comparison to next-generation sequencing methods and can be used to address a large variety of research questions. PacBio single-molecule real-time (SMRT) isoform sequencing (Iso-Seq) can capture the full length of transcripts, thereby presenting an easier and more accurate method for gene annotation [3], isoform identification [4, 5], and lncRNA discovery [6]. Thus, it has been successively used for whole-transcriptome profiling in many different organisms [7–9]. On the other hand, ONT sequencers measure changes in ionic current when the DNA fragments translocate through protein nanopores in a semi-synthetic insulated membrane; this process does not require enzyme-based nucleotide incorporation or detection of fluorescence signals. Therefore, the sequencing read length is theoretically limited only by the length of the DNA fragment translocating through the pore, and amplification biases can be avoided. For these reasons, ONT sequencing has recently allowed researchers to produce high-quality whole-genome assemblies of species such as humans [10], *Arabidopsis thaliana* [11], and *Solanum pennellii* [12]. Increasing studies are utilising ONT sequencers for transcriptome sequencing in humans and animals; however, studies of plants are limited.

In this study, we conducted a comprehensive comparison of PacBio and ONT applications in plant transcriptome sequencing, including read length, error rate, error pattern, coding region (CDS) and lncRNA prediction, complex transcriptome event discovery, and transcript abundance using ONT. This work provides a valuable reference for applications of ONT in plant transcriptome analysis.

## Results

### Overview of Illumina, PacBio and ONT sequencing

To compare the performance of the RNA sequencing methods, we sequenced cDNA libraries from *Arabidopsis* on Illumina NovaSeq, PacBio Sequel, Nanopore instruments. In addition, using Oxford nanopore sequencing, we sequenced cDNA directly (ONT Dc) and amplified cDNA (ONT Pc) using Nanopore GridION and Nanopore PromethION, respectively.

After sequencing, we obtained more than 21 million clean reads from each Illumina RNA-Seq replicate (Additional file 1: Table S1). The clean reads of each replicate were mapped to the reference genome; the percent of total mapped reads was > 84.67% (Additional file 1: Table S2).

**Fig. 1:**
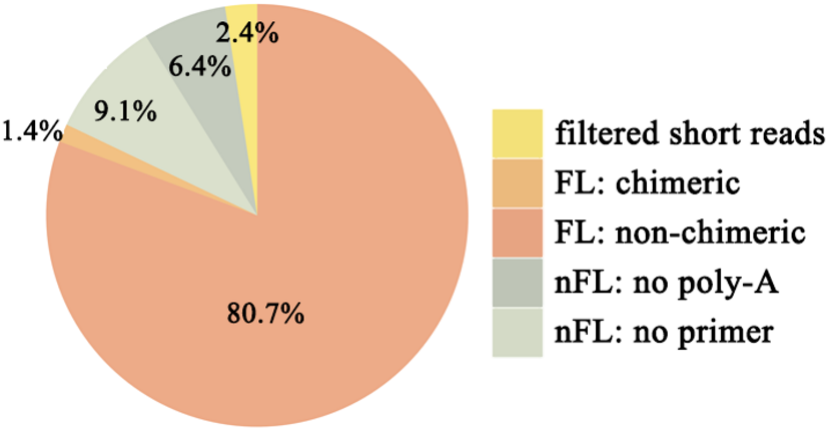
Classification of ROIs

For PacBio SMRT sequencing, one size-fractionated, full-length cDNA library (1–6 kb) was constructed and subsequently sequenced in one SMRT cell. As a result, we obtained 26.71 Gb of clean data. With full passes ≥ 0.8 and a predicted consensus accuracy > 0.80, 516,364 ROIs were successfully extracted with 27 passes and with a mean length of 1799 bp and quality of 0.97 (Additional file 1: Table S3). These ROIs included 416,662 (80.7%) full-length non-chimeric (FLNC) and 79,984 (15.5%) non-full-length (nFL) reads (Fig. 1). Then, using the ICE algorithm for clustering, we finally obtained 181,135 consensus isoforms in *Arabidopsis*.

For ONT Dc sequencing, we obtained 6,892,169, 5,687,972, and 10,936,056 clean reads from CTRL1, CTRL2, and CTRL3, respectively. The N50 values of these reads were 1245, 1438, and 1345, and the mean lengths were 1065, 1228, and 1167, respectively. Then, full-length sequences were identified if primers were found at both ends. As a result, we obtained 128,781, 138,295, and 262,832 full-length reads (FL reads) from CTRL1, CTRL2, and CTRL3, respectively (Additional file 1: Table S4). For ONT Pc sequencing, 8,146,264, 7,713,840, and 6,912,956 clean reads were obtained from CTRL1, CTRL2, and CTRL3, respectively. The N50 values of these reads were 1252, 1292, and 1270 and the mean lengths were 1222, 1246, and 1225, respectively. Among them, 5,682,227, 5,563,209, and 5,207,164 FL reads were obtained from CTRL1, CTRL2, and CTRL3, respectively (Additional file 1: Table S4).

### Comparison of raw data from PacBio and ONT results

To compare the raw data from PacBio and ONT sequencing, we randomly selected 10 Mb raw reads (3,112,439 subreads) from PacBio and 100,000 1D reads from each ONT sample (300,000 total).

The read length is a good representation of the useful length of long reads. The mean length of PacBio reads was 1410.186 bp, with median and maximum read lengths of 1302 bp and 89,075 bp respectively. ONT Dc data were shorter, with median and maximum lengths of 771 bp and 61,315 bp, respectively. The median and maximum read lengths of ONT Pc data were 1097 bp and 8236 bp, respectively (Table 1).

**Table 1:** Read length distribution of PacBio and ONT raw data

Sequencing type	Read length		
	Mean	Median	Maximum
PacBio	1410.186	1302	89075
ONT Dc	902.0619	771	61315
ONT Pc	1231.308	1097	8236

**Fig. 2:**
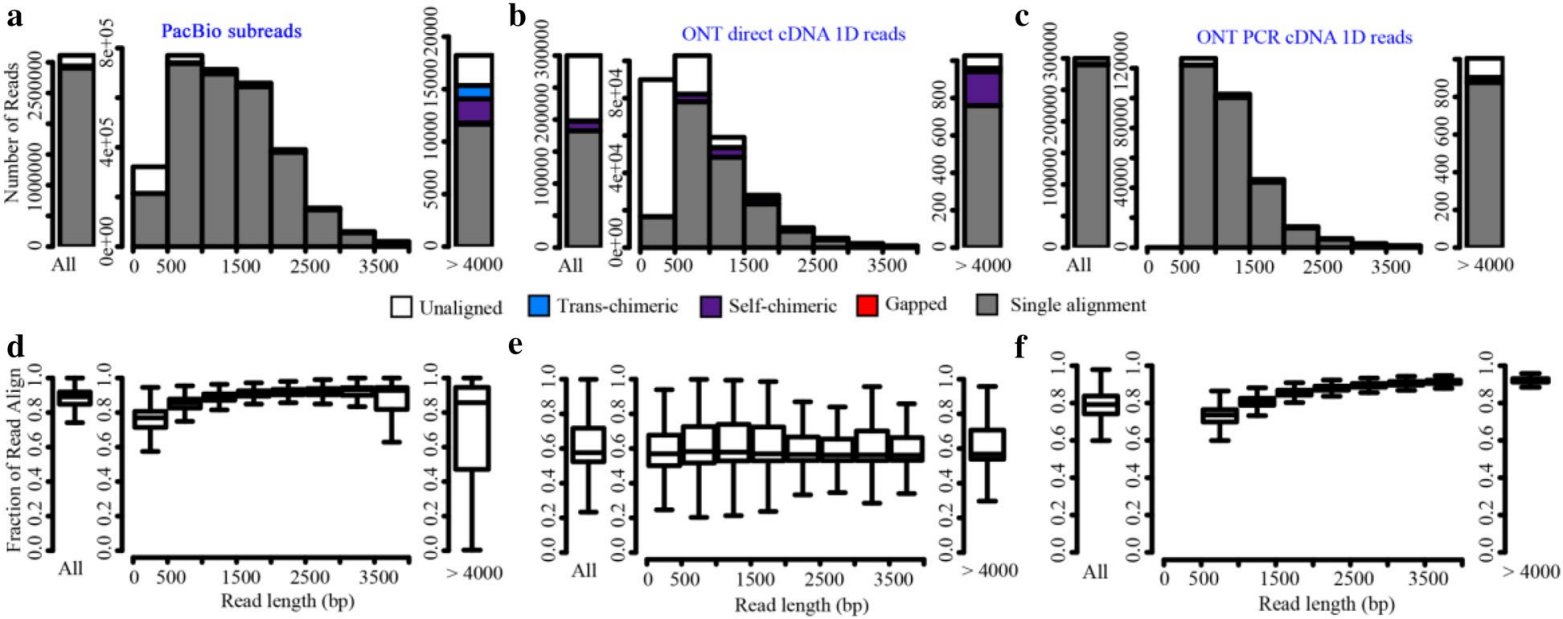
Length distributions and mappability of reads. **a–c** the length distributions of PacBio subreads (**a**), ONT Dc 1D reads (**b**), and ONT Pc 1D reads (**c**). Aligned reads are colour coded to indicate the fractions of reads that are unaligned (white), trans-chimeric alignments (blue), self-chimeric alignments (purple), gapped alignments consisting of multiple paths (red), and single best alignments (grey).The leftmost bar represents all reads, the middle portion represents reads from 0 to 4 kb in length, and the rightmost represents reads > 4 kb in length. **d–f** the mappability of different length bins of PacBio subreads (**d**), ONT Dc 1D reads (**e**), and ONT Pc 1D reads (**f**). The leftmost bar represents the fraction of the mappable read length of the total read length for all reads. The middle section shows the mappable fraction for read lengths ranging from 0 to 4 kb in 500-bp increments, and the rightmost bar represents the mappable fraction of reads > 4 kb

The overall length distributions of the reads for both PacBio and ONT exhibited remarkable differences (Fig. 2a–c). Compared to PacBio, the length distribution of ONT Dc data was skewed to the left, with a large proportion of reads < 2000 bp in length (Fig. 2a–c). In addition, the length distribution of ONT Pc data was similar to that of the ONT Dc data (Fig. 2b, c).

Mappability of long reads is essential for confirming repetitive elements, gene isoforms, and gene fusions. Of PacBio subreads, 94.5% were aligned to the reference genome (Fig. 2d, Table 2). Compared to the PacBio subreads, ONT Dc 1D reads had a lower rate (66%) (Fig. 2e, Table 2), and ONT Pc 1D reads had a higher rate of alignment (97%) (Fig. 2f, Table 2). For PacBio and ONT Pc data, we found that short read lengths (< 500 bp) had low alignment rates (Fig. 2d, f). This is likely due to a larger portion of adapter and linker sequences in this short-length data bin. However, of the ONT Dc 1D reads, all lengths had similar alignment rates (around 60%) (Fig. 2e).

**Table 2:** Alignment results of PacBio and ONT data

	PacBio	ONT Dc	ONT Pc
Total reads	3,112,439	300,000	300,000
Unaligned reads	170,385 (5.5%)	101,892 (34.0%)	8909 (3.0%)
Aligned reads	2,942,054 (94.5%)	198,108 (66.0%)	291,091 (97.0%)
Single-align reads	2,904,280 (93.3%)	182,478 (60.8%)	289,504 (96.5%)
Gapped-align reads	529 (0.02%)	237 (0.08%)	50 (0.02%)
Chimeric reads	37,245 (1.20%)	15,393 (5.13%)	1,537 (0.51%)
Trans-chimeric reads	22,921 (0.74%)	710 (0.24%)	1,296 (0.43%)
Self-chimeric reads	14,324 (0.46%)	14,683 (4.89%)	241 (0.08%)

Some regions of long reads may be particularly error prone, and long reads may be aligned as separated fragments, called gap-aligned reads. Corresponding to the high error rate, more ONT Dc data were gapped alignments (0.08%) compared to PacBio subreads (0.02%) and ONT Pc 1D reads (0.02%) (Table 2). These rates are very low and can be considered negligible in third-generation sequencing data. Long reads generated from gene fusions or trans-splices can be aligned to separate genomic loci; these are termed “trans-chimeric reads”. PacBio subreads contained 0.74% trans-chimeric reads, whereas ONT 1D data contained fewer (ONT Dc: 0.24% and ONT Pc: 0.43%) (Table 2). In addition, the PacBio subreads showed notably higher trans-chimeric rates in very long reads (> 4 kb) (Fig. 2a). Two fragments of a long read may be aligned to the same genomic locus, termed “self-chimeric”, because of the failure to remove adaptor sequences from the raw data. PacBio subreads and ONT Pc 1D reads contained 0.46% and 0.08% self-chimeric reads, respectively, while ONT Dc 1D reads had a surprisingly higher rate (4.89%) (Table 2). The chimeric reads may cause overestimation of DNA molecule lengths.

Error rates and error patterns can indicate the quality of the data, which has a strong effect on single-nucleotide resolution analysis. The error rate of PacBio was 13.217%. Compared to the PacBio subreads, the error rate of ONT Dc data was slightly higher, reaching 13.934%, while the error rate of ONT Pc data was lower (12.669%) (Table 3). This indicated that the ONT Pc data were of slightly higher base quality than PacBio data.

**Table 3:** Error pattern of PacBio and ONT data

	PacBio	ONT Dc	ONT Pc
Bases analyzed	1,117,976	1,066,943	1,079,480
Correctly aligned bases	970,213 (86.8%)	917,270 (86.1%)	942,722 (87.3%)
Total error bases	147,7963 (13.217%)	148,673 (13.934%)	136,758 (12.669%)
Mismatched bases	45,654 (4.084%)	50,249 (4.710%)	46,978 (4.352%)
Deletion bases	58,194 (5.205%)	62,423 (5.851%)	54,896 (5.085%)
Insertion bases	43,915 (3.928%)	36,001 (3.374%)	34,884 (3.232%)

In addition, the compositions of PacBio and ONT errors were similar. The proportions of mismatches were 4.084%, 4.710%, and 4.352% for PacBio, ONT Dc, and ONT Pc data, respectively (Table 3). The deletions had higher rates, 5.205%, 5.851%, and 5.085%, in PacBio, ONT Dc, and ONT Pc data, respectively; the insertion rates were 3.928%, 3.374%, and 3.232%. Taken together, the deletions and insertions together (indels) contributed the most errors in both PacBio and ONT data.

**Fig. 3:**
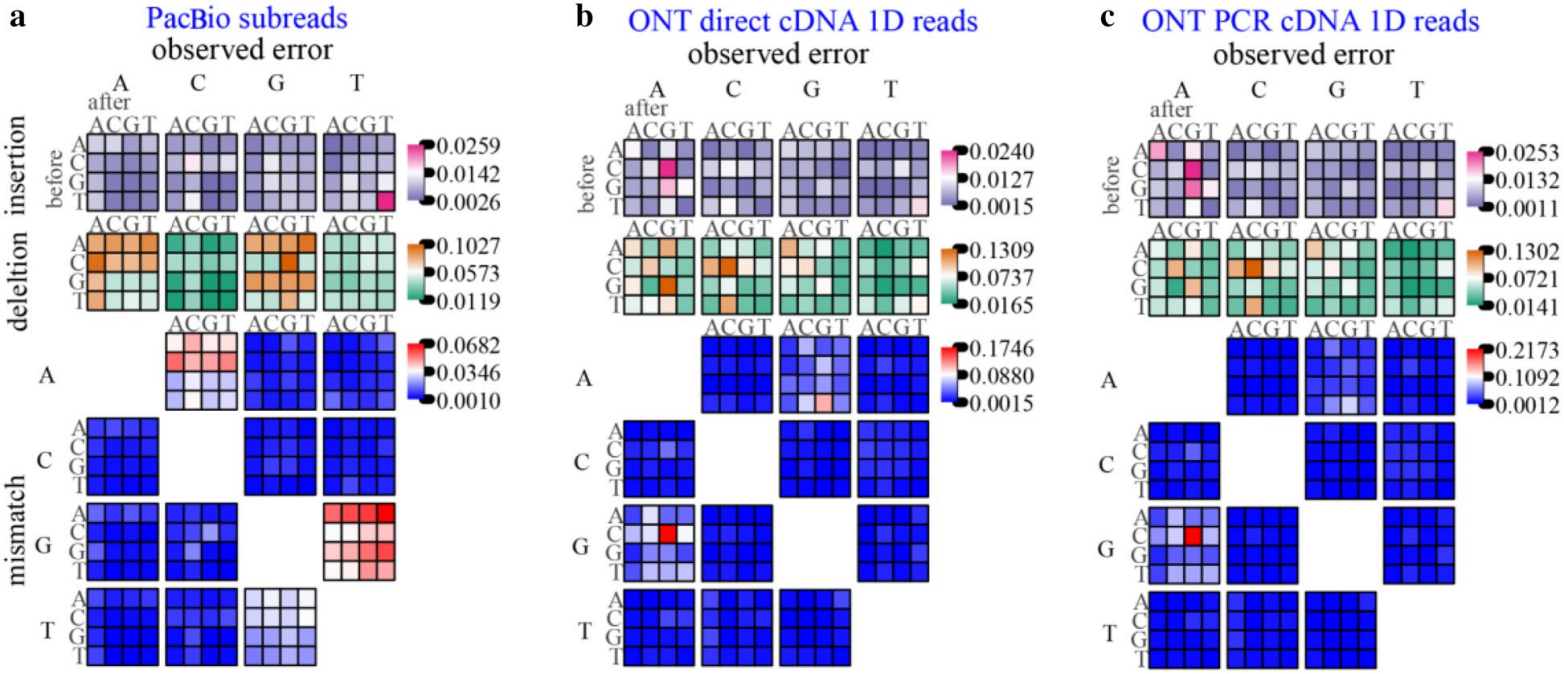
Context-specific errors. Context-specific errors of **a** PacBio subreads, **b** ONT Dc 1D reads, and **c** ONT Pc 1D reads. The error types shown are insertions, deletions, and mismatches. For insertions (deletions), the large base above the plot indicates the inserted (deleted) base. For mismatch errors, the large base to the left (above) indicates the expected (observed) base. Blocks of coloured tiles show the error frequency in specific contexts for each error; the small base to the left (above) indicates the base preceding (following) the error. Error frequency is plotted on separate scales for insertions, deletions, and mismatches

Both PacBio and ONT errors exhibited context-specific patterns. In PacBio reads, most mismatches arose from several context-specific events such as GA → TA, GC → TC, GG → TG, and GT → TT (Fig. 3a). The mismatch CG → CA was most abundant in ONT Dc data, followed by AG → GG (Fig. 3b). The context-specific mismatches in ONT Pc data were similar to those in ONT Dc data, with CG → CA as the most abundant (Fig. 3c). In addition, T in insertions and A and G in deletions were most commonly observed in PacBio reads (Fig. 3a), whereas A in insertions and A and C in deletions were most common in ONT data (Fig. 3b, c).

### Transcriptome construction from Illumina, PacBio, and ONT data

Based on Illumina short reads, a total of 21,157 genes were obtained. For PacBio sequencing, the consensus isoforms were polished using non-full-length reads, and 129,080 transcripts with high quality were obtained. These corrected transcripts were mapped to the reference genome using GMAP software. After removal of redundant reads, 38,011 non-redundant mapped transcripts from 13,376 gene loci were generated. For ONT Dc sequencing, after polishing all of the full-length reads, the corrected isoforms were mapped against the reference genome, generating a total of 47,601 unique mapped transcripts. Similarly, ONT Pc sequencing generated 36,775 non-redundant mapped transcripts.

**Fig. 4:**
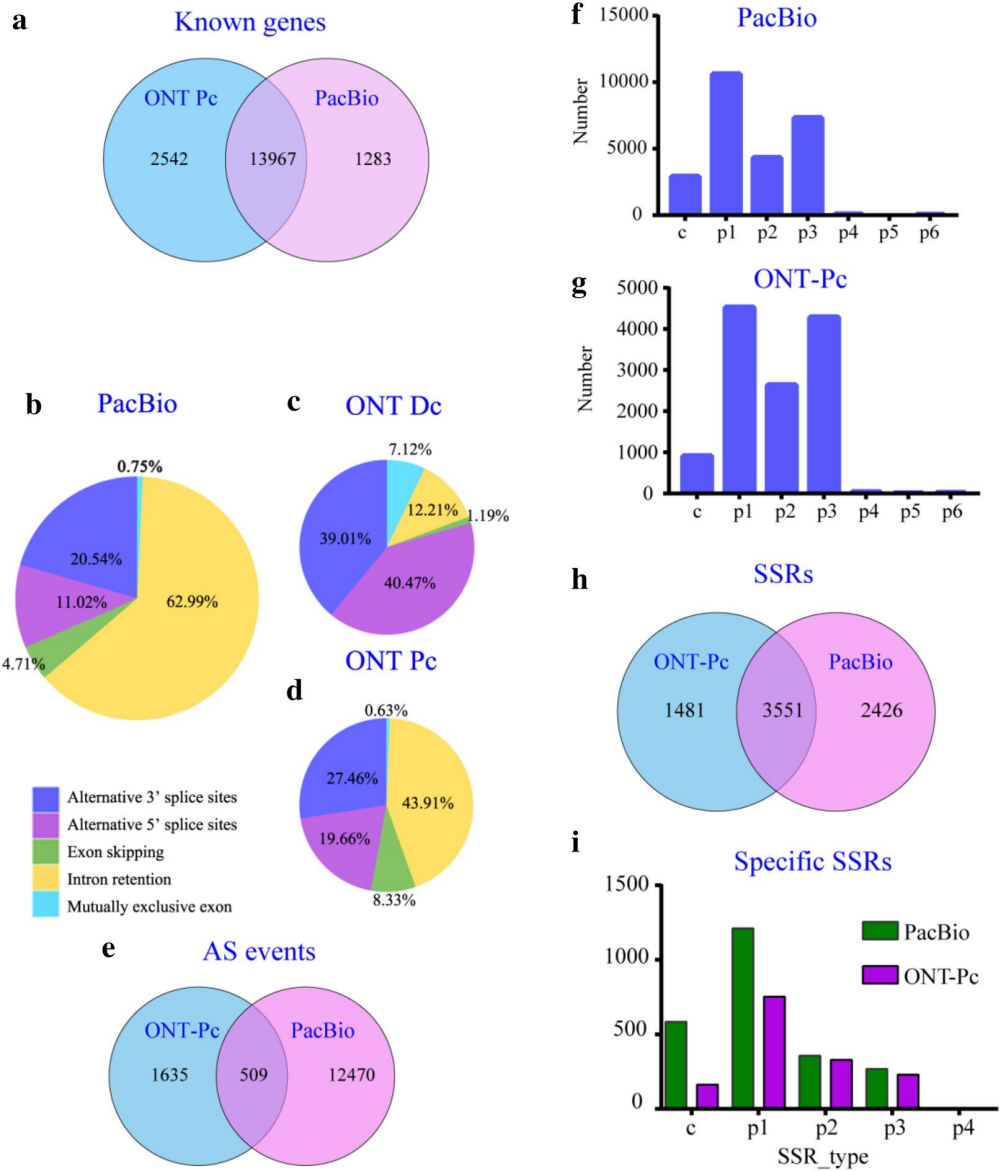
Multiple analyses of genes, AS events and SSRs. **a** Venn chart showing the numbers of known genes identified in PacBio and ONT Pc. **b**–**d** Alternative splicing event distribution of PacBio (**b**) ONT Dc (**c**) and ONT Pc (**d**) data. **e** Venn chart showing the numbers of AS events detected in PacBio and ONT Pc. **f**–**g** Distribution of different SSR type from PacBio (**f**) and ONT Pc (**g**) technologies. **h** Venn chart showing the numbers of SSR screened in PacBio and ONT Pc. **i** Distribution of different SSR type of specific SSR identifying in PacBio (green) and ONT Pc (purple). c, compound SSR, the length beween the different SSR < 100 bp; p1, Mono-nucleotide repeat; p2, Di-nucleotide repeat; p3, Tri-nucleotide repeat; p4, Tetra-nucleotide repeat; p5, Penta-nucleotide repeat; p6, Hexa-nucleotide repeat

We first compared the transcript lengths among the different sequencing technologies. The mean length of PacBio transcripts was 2072.57 bp; the median and maximum read lengths were 1988 bp and 8105, respectively. ONT Dc data were shorter, with median and maximum lengths of 1249.97 bp and 6763 bp. The median and maximum read lengths of ONT Pc data were 1332 bp and 7319 bp, respectively (Table 4). Among the identified known genes, 13,967 genes were commonly identified by ONT Pc and PacBio. Moreover, 2,542 and 1283 known genes were specifically identified by ONT Pc and PacBio, respectively (Fig. 4a). Thus, ONT showed a superior performance in known gene identification over PacBio. Furthermore, 1283 specific transcripts in PacBio displayed a median length of 3305 and 39% GC content; while the 2542 specific transcripts in ONT Pc showed a median length of 1565 and 38% GC content (Additional file 1: Table S5). These results indicate the suitability of the ONT-Pc for finding relatively small full length transcripts.

**Table 4:** Read length distribution of PacBio and ONT identified transcripts

Sequencing type	Read length		
	Mean	Median	Maximum
PacBio	2072.57	1988	8105
ONT Dc	1249.97	1135	6763
ONT Pc	1474.28	1332	7319

### AS events

Within the PacBio unique mapped reads, we detected a total of 12,979 AS events, including 97 mutually exclusive exon events, 8175 intron retention (IR) events, 611 exon-skipping (ES) events, 1430 alternative 5′ sites (Alt. 5′), and 2666 alternative 3′ sites (Alt. 3′). In PacBio data, the most frequent AS events identified were IR events (62.99%), followed by Alt. 3′ (20.54%), Alt. 5′ (11.02%), and ES events (4.71%); few mutually exclusive exon events (0.75%) were discovered (Fig. 4b).

Far fewer AS events were detected in ONT Dc data; CTRL1, CTRL2, and CTRL3 contained 1433, 928, and 4367 AS events, respectively. The fractions of each AS type also differed from the PacBio data. The most identified AS events in ONT data were Alt. 3′ events, followed by Alt. 5′, IR, and mutually exclusive exon events; the fewest were ES events (1.07%) (Fig. 4c). By contrast, in the ONT Pc data, 1897, 2048, and 2034 AS events were identified in CTRL1, CTRL2, and CTRL3, respectively. The fractions of each AS type were similar to those of the PacBio data (Fig. 4d). There were only 509 common AS events in both PacBio and ONT Pc (Fig. 4e), including 170 Alt. 3′ events (33.40%), 62 Alt. 5′ events (12.18%), 44 ES events (8.64%) and 233 IR events (45.78%) (Additional file 1: Table S6). The results showed that ONT Pc has a relative weakness in AS event detection.

### SSR detection

Transcripts  > 500 bp in length were selected for SSR analysis using MISA. A total of 58,885 sequences (122,942,629 bp) were subjected to SSR analysis. As a result, we identified a total of 29,394 SSRs and 20,243 SS-containing sequences from PacBio data (Additional file 1: Table S7). There were 6067 sequences containing more than one SSR, and 4,109 SSRs were present in compound formation. Furthermore, the repeat units of SSR loci are 1 ~ 6 bases, in which Mono-nucleotide repeats (p1) were the most (10623, 42.01%), followed by Tri-nucleotide repeats (p3: 7316, 28.93%) and Di-nucleotide repeats (p2: 25285. 17.10%). The 4 base and more repeat units are relatively less (Fig. 4f).

Within ONT Dc data, 32,854 sequences of  > 500 bp (53,964,961) were used for SSR analysis. A total of 9,234 SSRs and 7543 SSR-containing sequences were identified. Similarly, in the ONT Pc data, 35,305 transcripts (53,588,806 bp) contained 13,415 SSRs and 10,350 SSR-containing sequences (Additional file 1: Table S7), in which Mono-nucleotide repeats  > Tri-nucleotide repeats  > Di-nucleotide repeats, and accounted for 36.45%, 34.54% and 21.23%, respectively. And it is similar to PacBio that the 4 ~ 6 base repeat units were relatively less (Fig. 4g). Furthermore, a total of 3551 SSRs were commonly identified by ONT Pc and PacBio (Fig. 4h). Moreover, 2426 and 1481 SSRs were specifically identified by PacBio and ONT Pc, respectively. Among the specific SSRs, PacBio and ONT Pc exhibited similar ability to identify 2 ~ 4 base repeat units, while PacBio showed superior performance in Mono-nucleotide repeats and compound SSR identification (Fig. 4i).

### CDSs of new transcripts and lncRNA prediction

**Fig. 5:**
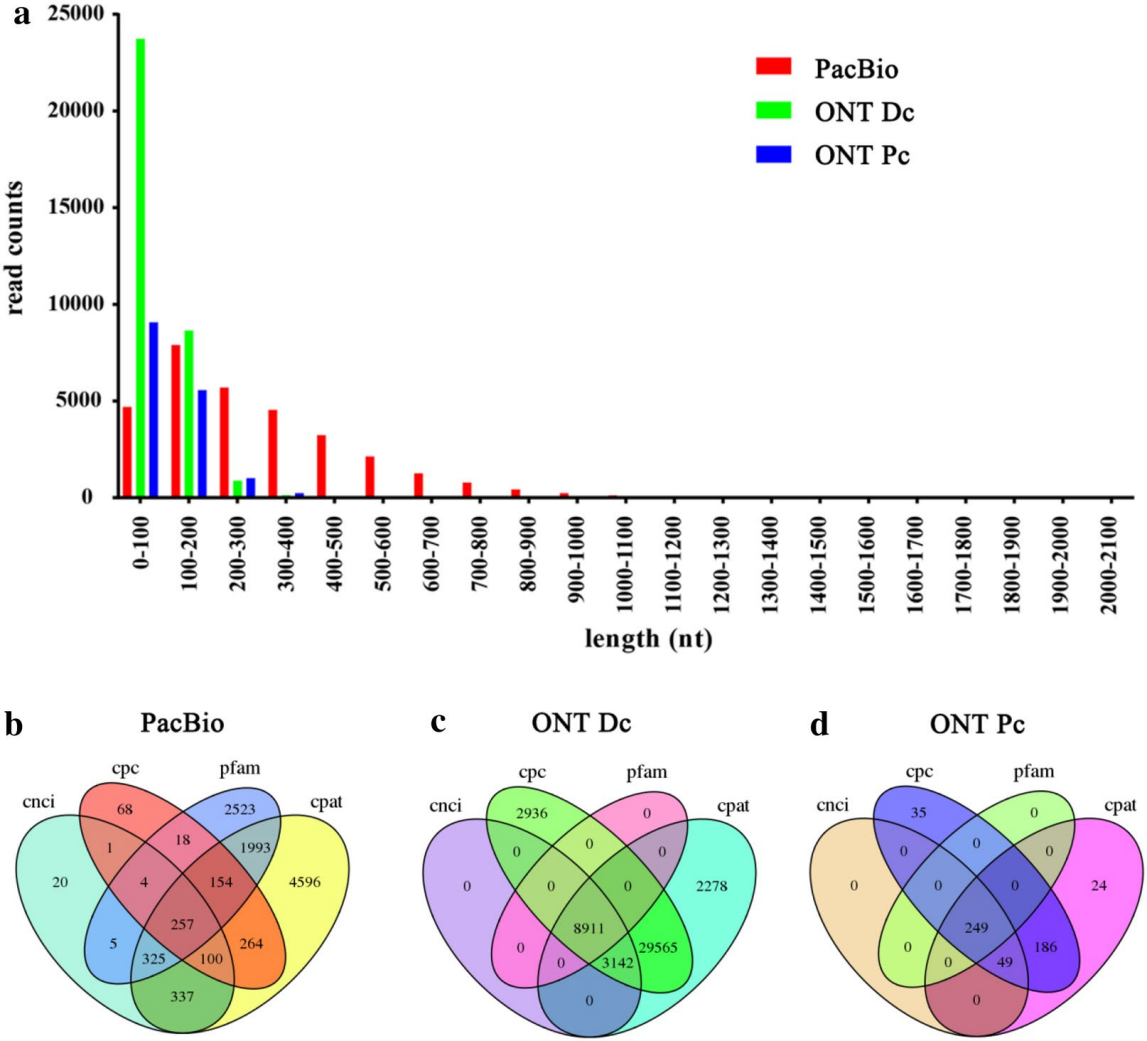
Length distributions of complete ORFs and identification of lncRNAs. **a** length distributions of the CDS lengths of complete ORFs in PacBio, ONT Dc, and ONT Pc results, **b**–**d** venn diagrams showing the numbers of candidate lncRNAs screened using Pfam, CPC, CNCI, and CPAT for PacBio (**b**), ONT Dc (**c**), and ONT Pc (**d**) results

Using TransDecoder (v3.0.0), 31,137 ORFs were identified in the PacBio data, of which 25,256 were complete ORFs. In addition, 33,419 and 15,968 complete ORFs were predicted in the ONT Dc and ONT Pc data, respectively. Figure 5a shows the length distribution of the CDSs of complete ORFs. In the PacBio data, the CDS lengths of complete ORFs mostly ranged from 100 to 1000 bp (Fig. 5a). However, the length distribution was skewed to the left in the ONT Dc and ONT Pc data. In the ONT Dc data, most CDS lengths of complete ORFs were 0–100 bp, followed by 100–200 bp, and only a few reads were  > 200 bp. Similarly, in the ONT Pc data, the lengths of CDS of complete ORFs ranged from 0 to 800 bp, with most being 0–300 bp (Fig. 5a).

**Fig. 6:**
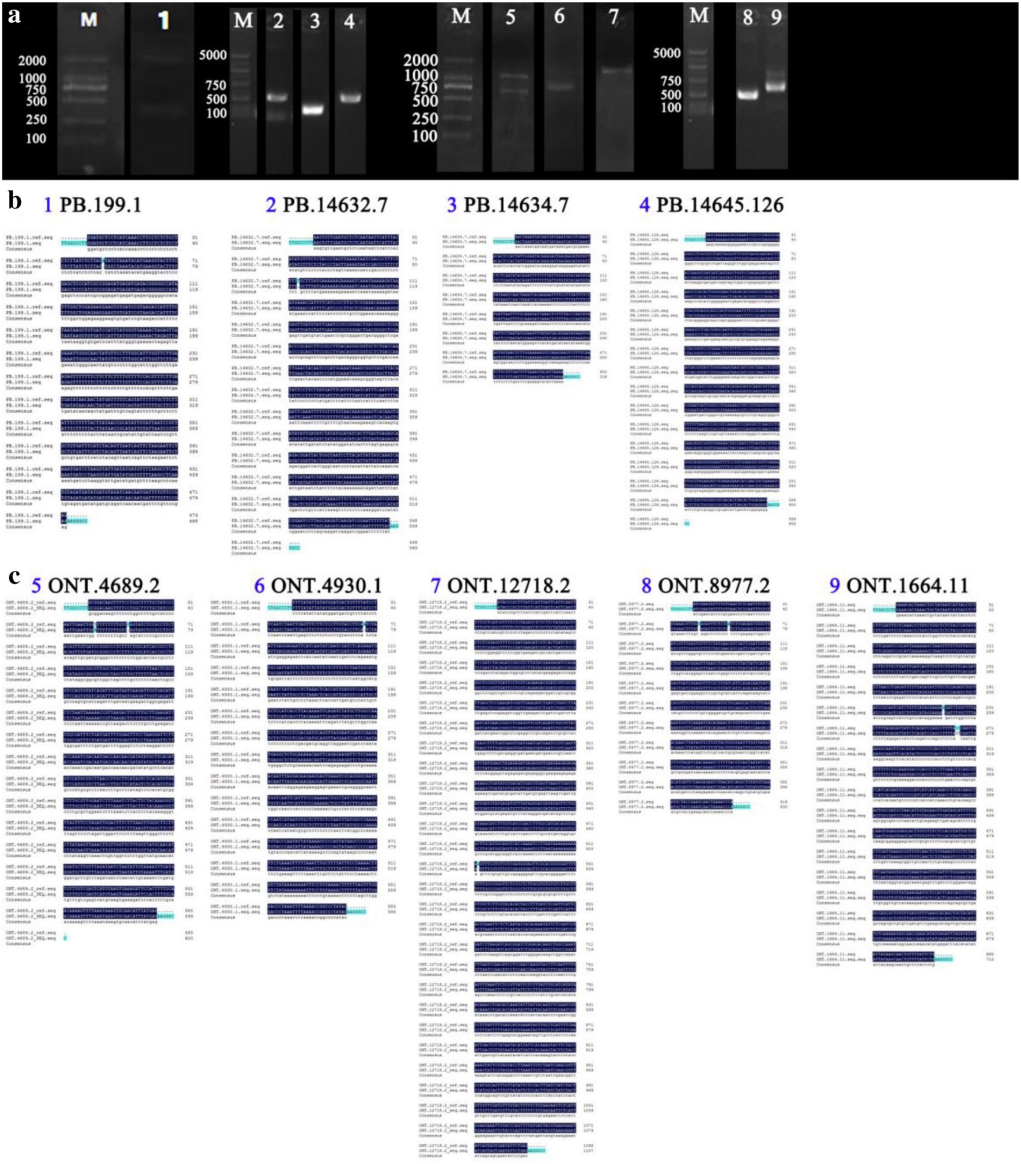
Sequence validation of predicted lncRNAs. **a** PCR products of randomly selected lncRNAs, **b** alignment between Sanger sequencing results and PacBio sequencing results for identified lncRNAs. **c** Alignment between Sanger sequencing results and ONT Pc sequencing results for identified lncRNAs

Using CPC, CNCI, Pfam protein structure domain analysis, and CPAT, totals of 257, 8911, and 249 lncRNAs were predicted by all four methods from PacBio, ONT Dc, and ONT Pc data, respectively (Fig. 5b–d). Thirty-five common lncRNAs were identified both in PacBio and ONT Pc data (Additional file 1: Table S8). We randomly selected 16 unique lncRNAs (8 lncRNAs from PacBio data and 8 lncRNAs from ONT Pc data) for validation by PCR amplification and Sanger sequencing. Of the 8 lncRNAs from PacBio data, 2 were completely identical to the RNA-Seq sequences, and 2 had fewer than three mismatched nucleotides (Fig. 6a, b). In addition, of the eight selected lncRNAs from ONT Pc data, 5 lncRNAs were verified, all of which had fewer than three mismatched nucleotides (Fig. 6a, c).

### Isoform abundance estimation by ONT and Illumina data

**Fig. 7:**
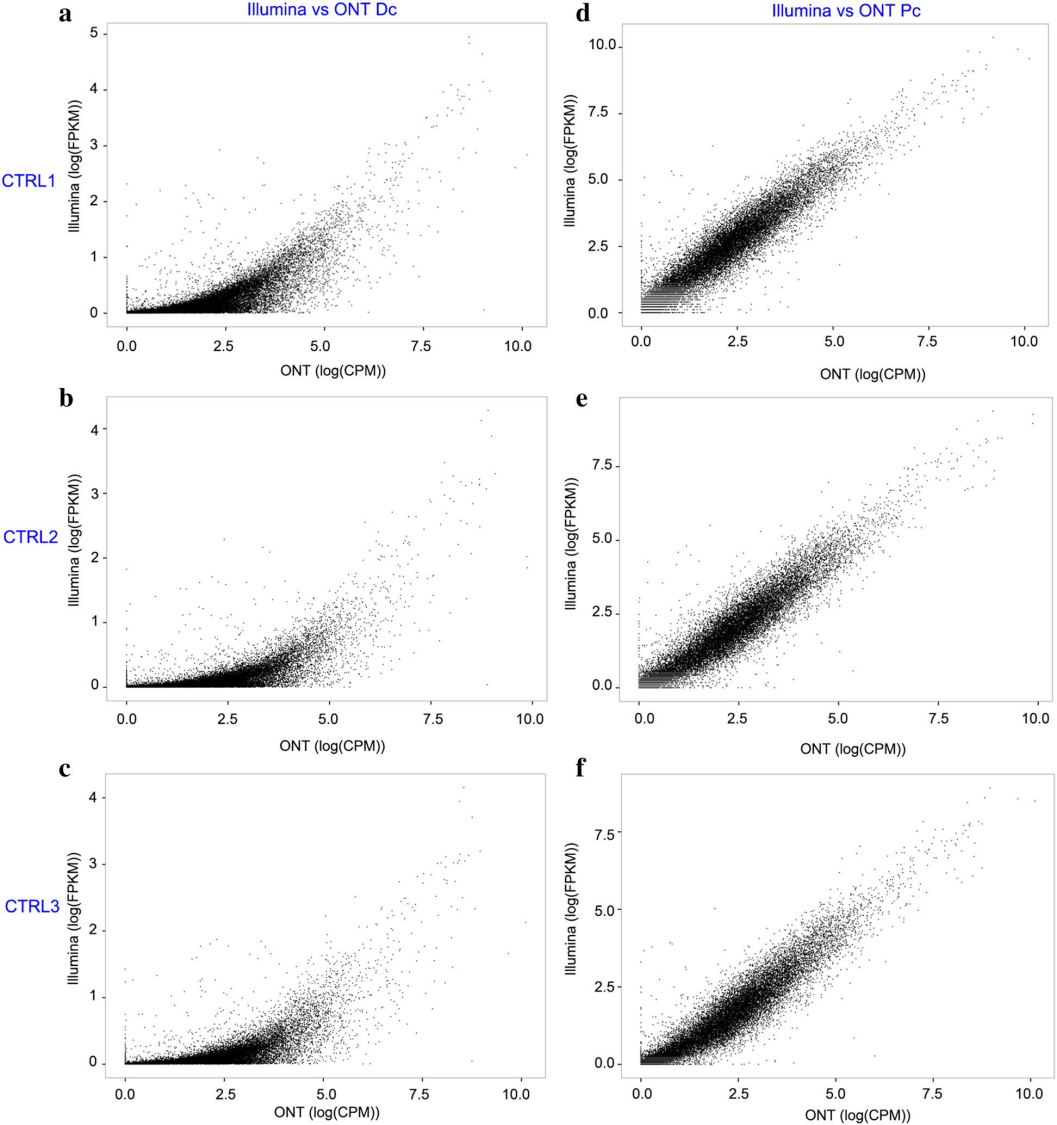
Transcript expression correlations between Illumina and ONT results. **a–c** expression correlations between Illumina and ONT Dc results in CTRL1, CTRL2, and CTRL3. **d–f** expression correlations between Illumina and ONT Pc results in CTRL1, CTRL2, and CTRL3

We evaluated the performances of ONT and Illumina data on transcript quantification. Fragments per kilobase of transcript per million fragments mapped (FPKM), and counts per million (CPM) values were used to quantify transcript expression levels of Illumina (Additional file 1: Table S9), ONT Dc (Additional file 1: Table S10) and ONT Pc (Additional file 1: Table S11) data, respectively. We further calculated the correlation between Illumina and ONT Dc data of each repeat. The results showed that the expression correlation values between Illumina and ONT Dc data of CTRL1, CTRL2, and CTRL3 were 0.747, 0.719, and 0.711, respectively (Fig. 7a–c). The expression correlation values between Illumina and ONT Pc were higher, at 0.932, 0.928, and 0.923 for CTRL1, CTRL2, and CTRL3, respectively (Fig. 7d–f).

## Discussion

Next-generation sequencing is still the primary technology used for transcriptome studies. However, short-read RNA-Seq methods are limited in their ability to identify complex transcript isoforms because they cannot sequence full-length transcripts. Furthermore, transcripts are fragmented, which results in short individual reads that fail to span the entirety of the transcripts. To overcome the limitations of short-read RNA-Seq, TGS, including single-molecule long-read PacBio Iso-Seq and ONT, was developed. To date, many studies have successfully used PacBio Iso-Seq to sequence full-length cDNA samples derived from both animals and plants [7, 13]. In recent years, ONT has also been used to analyse full-length cDNA samples from mouse individual B cells [14] and human embryonic stem cells [15]. In practice, PacBio and ONT sequencing have their own merits and demerits. Briefly, PacBio sequencers produce low numbers of reads with high accuracy, while ONT sequencers produce higher numbers of reads with lower accuracy. Furthermore, PacBio Iso-Seq is commercially expensive, and ONT sequencing is more cost-effective. Thus, in the present study, we performed a detailed evaluation of reads from PacBio and Nanopore direct cDNA and PCR cDNA sequencing in plants (*Arabidopsis*) based on the characteristics of raw data and identification of transcripts. In addition, matched Illumina data were generated for comparison of transcript expression between ONT and Illumina. We aimed to select a method to obtain long stretches of sequences at bargain basement prices.

Overall, we observed that PacBio and ONT can similarly generate long read lengths with relatively high error rates. However, they have some differences in their raw data. Because the quality of ONT Dc sequencing was far below that of ONT Pc sequencing, we focused on the comparison between the results of PacBio and ONT Pc sequencing. First, among the randomly selected raw data, the maximum read length of PacBio results was 89,075 bp, whereas that of ONT Dc data was 61,315 bp. Unexpectedly, the maximum read length of ONT Pc data was notably shorter than those of PacBio and ONT Dc, at only 8236 bp. Second, previous studies have shown that PacBio can generate higher quality raw data with a lower error rate and higher mappability compared to ONT raw data [15]. In our study, the mappability of ONT Pc data was 97.0%, whereas that of PacBio raw data was 94.5%. In addition, the error rate of PacBio was 13.217%, and the ONT error rate was slightly lower at 12.669%. Thus, our results indicated that ONT Pc data were of a higher quality, suggesting that ONT may be a promising tool for transcriptome-wide studies.

TGS methods have been applied in sequencing of animals and humans and have proven superior to short-read sequencing methods due to the advantage of obtaining full-length transcripts [16]. Thus, they can be used to identify complex AS events, which can greatly increase transcriptome diversity. AS events include five different types: mutually exclusive exons, IR, ES, Alt. 5′, and Alt. 3′. Many studies have used long reads from PacBio and ONT to detect AS events. For example, Iso-Seq identified 10,053 and 21,154 AS events in *Sorghum bicolor* [4] and *Phyllostachys edulis* [5], respectively. In our study, PacBio identified 12,979 AS events, whereas ONT Pc only identified ~ 2000 AS events from each sample. The number of AS events identified was much higher in the PacBio data, indicating that PacBio sequencing results may serve as a more valuable resource for the study of transcriptome complexity and gene regulation.

LncRNAs are defined as RNAs that are > 200 nt in length and have no coding potential [17]. Most lncRNAs are polyadenylated in plants, so Illumina RNA-Seq can both detect lncRNAs and quantify their expression. However, recently, several studies have reported that lncRNAs undergo complex post-transcriptional regulation [18], and full-length sequencing showed great advantages in identifying gene models of lncRNAs. Iso-Seq in poplar and *Trifolium pratense* L. revealed 1187 and 4333 lncRNAs, respectively [19, 20], suggesting that Iso-Seq is a suitable method for identification of lncRNAs. Here, we identified 257 and 249 lncRNAs based on PacBio and ONT Pc data. Furthermore, PCR and Sanger sequencing validated four of eight and five of eight lncRNAs from the PacBio and ONT Pc data, respectively. These results suggest that both ONT Pc and PacBio methods are suitable for identification of lncRNAs.

Compared to PacBio sequencing, one of the greatest advantages of ONT is that it can estimate transcript expression levels [21]. In the present study, we analysed the correlation between Illumina and ONT Dc data of each replicate sample and found correlations > 0.8 for all groups. The high correlation suggests that ONT can well quantify transcript expression levels.

## Conclusions

In conclusion, our results showed that ONT Pc performed well in transcript identification, SSR analysis, and lncRNA prediction. ONT Pc generated better quality data in terms of error rate and mappability, and PacBio generated longer sequence reads. Although PacBio is superior in identifying AS events, ONT Pc can quantify transcripts of different lengths. In addition, ONT is less expensive than PacBio. Taken together, these results indicate that ONT Pc is more cost-effective for generating extremely long reads and can characterise the transcriptome as well as quantify transcript expression. Therefore, it is a good choice for full-length single-molecule transcriptome analysis in plants.

## Methods

Seeds of *A. thaliana* [wild-type Columbia (Col-0)] were exposed to stratification for 2 d at 4 °C, then sown in square surface-sterilised plastic pots (7 cm × 7 cm × 8 cm) containing sterile medium [1:1 (v/v) mixture of vermiculite and peat]. Pots were arranged in a plastic pallet and placed in a growth chamber (23 °C during the day and 18 °C at night, with a 16-h photoperiod and 500 μmol m^−2^ s^−1^ of photosynthetically active radiation); the plants were watered to saturation with, alternately, distilled water or 1/2 Murashige–Skoog solution [22]. The aboveground parts were collected at a growth stage of 3.90 (rosette growth complete). Each replication contained 15–20 plants.

Total RNA was extracted using the RNAprep Pure Plant Kit (Tiangen, Beijing, China). The RNA Nano 6000 Assay Kit of the Agilent Bioanalyzer 2100 system (Agilent Technologies, Santa Clara, CA, USA) was used to assess RNA integrity, and the Qubit RNA Assay Kit and Qubit 2.0 Fluorometer (Life Technologies, Carlsbad, CA, USA) were used to quantify the extracted RNA.

### Library preparation and sequencing

For Illumina sequencing, cDNA libraries (with three biological replicates) were constructed using the NEBNext Ultra RNA Library Prep Kit for Illumina (New England Biolabs, Beverly, MA, USA) following the manufacturer’s protocol. The libraries were sequenced on an Illumina NovaSeq platform, and paired-end reads were generated.

For PacBio sequencing, cDNA from the same RNA samples used for Illumina sequencing was synthesised using the SMARTer PCR cDNA Synthesis Kit. After PCR amplification, products were sequenced on the PacBio Sequel platform.

For ONT sequencing, cDNA-PCR libraries were built using the Ligation Sequencing Kit (SQK-LSK109) and sequenced on the Nanopore PromethION platform. Direct-cDNA libraries were built using the Direct cDNA Sequencing Kit (SQK-DCS108) and sequenced on the Nanopore GridION X5 platform.

### Comprehensive quality assessment for long-read sequencing

The AlignQC software (https://github.com/jason-weirather/AlignQC/wiki) was used to perform the comprehensive quality assessment for long-read sequencing. First, all data were uploaded into the software, and the memory was adjusted to 500 GB, however, the memory was insufficient. Therefore, we randomly selected 10 Mb raw reads from the PacBio results and 100,000 1D reads from each ONT sample for subsequent analyses. AlignQC accepted standard BAM format files as inputs and output XHTML format files for easy visualisation, providing links to access all analysis results.

### Transcript identification of PacBio long reads

The SMRT-Analysis software package v3.0 (https://github.com/ben-lerch/IsoSeq-3.0/blob/master/README.md) was used for Iso-Seq data analysis. First, reads of insert (ROIs) were generated using full passes ≥ 0.8. Then, with the examination of poly(A) signals and 5′ and 3′ adaptors, full-length and non-full-length cDNA reads were recognised. Consensus isoforms were identified by iterative clustering for error correction (ICE) algorithm and further polished to obtain high-quality consensus isoforms. Then the high-quality isoforms were mapped to the reference genome of *Arabidopsis*. Redundancy was removed from the mapped results using cDNA_Cupcake (https://github.com/Magdoll/cDNA_Cupcake/wiki). The reads with identity < 0.9 or coverage < 0.85 were filtered out, and reads in which only the 5′-end exons differed were combined. As a result, non-redundant transcripts were obtained.

### Transcript identification of ONT long reads

Nanopore sequencing raw data were base-called using the Guppy software in MinKNOW2.2. Then, short reads, low-quality reads, and reads with adaptors were filtered out to obtain the clean data. According to the principle of cDNA sequencing, a primer sequence identified at both ends of a read was indicative of a full-length sequence. Then, using minmap2 to map the read itself, overlap information between reads was obtained. Finally, consistent sequences were obtained using the Racon software. The high-quality isoforms were mapped to the reference genome of *Arabidopsis* using the Genomic Mapping and Alignment Program (GMAP). Redundancy was removed from the mapped results by same method used in the PacBio data analysis.

### SSR detection

Transcripts larger than 500 bp were selected for SSR analysis using the MIcroSAtellite identification tool (MISA). MISA can identify seven SSR types, including mono-nucleotide, di-nucleotide, tri-nucleotide, tetra-nucleotide, penta-nucleotide, hexa-nucleotide, and compound SSRs.

### Coding sequence prediction of new genes

The coding sequences and corresponding amino acid sequences within the transcript sequences were predicted by TransDecoder. TransDecoder can identify candidate protein-coding regions based on nucleotide composition, open reading frame (ORF) length, log-likelihood score, and optional protein family database (Pfam) domain content.

### lncRNA prediction

The transcripts with coding potential were filtered by the Coding Potential Calculator (CPC) [23], Coding–Non-Coding Index (CNCI) [24], Coding Potential Assessment Tool (CPAT) [25], and Pfam [26]. Finally, the remaining non-coding transcripts were selected as lncRNAs.

### Cloning and Sanger sequencing of lncRNAs

Total RNA was isolated from *Arabidopsis* as described above. cDNA was synthesised from 2 μg of purified total RNA using the PrimeScript 1st Strand cDNA Synthesis Kit (TaKaRa, Dalian, China) according to the manufacturer’s protocol. Sixteen pairs of primers were designed (Additional file 1: Table S12). PCR amplifications were carried out as follows: 94 °C for 5 min, followed by 35 cycles at 94 °C for 35 s, 55 °C or 58 °C for 15 s, and 72 °C for 50 s. Amplification products were separated on a 2% agarose gel. Gel-purified PCR fragments were cloned into the T5-simple Vector system (TransGen, Beijing, China) and sequenced.

### Quantification of gene expression levels

For Illumina sequencing, HISAT2 and StringTie were used for reads alignment and gene/transcript identification, respectively. And gene expression levels were estimated by fragments per kilobase of transcript per million fragments mapped (FPKM) using StringTie [27]. For ONT sequencing, Salmon was used for transcript counts estimation, and gene expression levels were estimated by counts per million (CPM) [28]. The formula is shown as follow: CPM = (reads mapped to transcript/total reads aligned in sample) × 1,000,000. The correlation between Illumina and ONT data was calculated using Pearson Correlation Coefficient.

## Supplementary information

**Additional file 1: Table S1**. Summary of clean data produced by Illumina sequencing of three repetitions. **Table S2**. Illumina reads mapped to the reference genome. **Table S3**. PacBio sequencing results. **Table S4**. Nanopore sequencing results. **Table S5**. Characteristics of PacBio and ONT Pc identified specific known genes. **Table S6**. Common AS events between PacBio and ONT Pc data. **Table S7**. SSR identification of PacBio and ONT data. **Table S8**. Common lncRNAs between PacBio and ONT Pc data. **Table S9**. Transcript expression levels of Illumina data. **Table S10**. Transcript expression levels of ONT Dc data. **Table S11**. Transcript expression levels of ONT Pc data. **Table S12**. Primer sequences for PCR validation.

## Data Availability

All raw sequence data have been deposited in the NCBI GSE 141641 (https://www.ncbi.nlm.nih.gov/geo/query/acc.cgi?acc=GSE141641).
